# [^111^In]In-CP04 as a novel cholecystokinin-2 receptor ligand with theranostic potential in patients with progressive or metastatic medullary thyroid cancer: final results of a GRAN-T-MTC Phase I clinical trial

**DOI:** 10.1007/s00259-022-05992-6

**Published:** 2022-11-05

**Authors:** Luka Lezaic, Paola Anna Erba, Clemens Decristoforo, Katja Zaletel, Renata Mikolajczak, Helmut Maecke, Theodosia Maina, Mark Konijnenberg, Petra Kolenc, Malgorzata Trofimiuk-Müldner, Elwira Przybylik-Mazurek, Irene Virgolini, Marion de Jong, Alide C Fröberg, Christine Rangger, Gianpaolo Di Santo, Konrad Skorkiewicz, Piotr Garnuszek, Bogdan Solnica, Berthold A. Nock, Danuta Fedak, Paulina Gaweda, Alicja Hubalewska-Dydejczyk

**Affiliations:** 1 University Medical Centre Ljubljana, Ljubljana, Slovenia; 2 Faculty of Medicine, University of Ljubljana, Ljubljana, Slovenia; 3 Regional Center of Nuclear Medicine, Department of Translational Research and New Technology in Medicine, University of Pisa, and Azienda Ospedaliero-Universitaria Pisana, Pisa, Italy; 4 Department of Nuclear Medicine, Medical University Innsbruck, Innsbruck, Austria; 5 National Centre for Nuclear Research, Radioisotope Centre POLATOM, Otwock-Świerk, Poland; 6 University Hospital Freiburg, Freiburg, Germany; 7 Molecular Radiopharmacy, INRASTES, NCSR Demokritos, Athens, Greece; 8 Erasmus MC, Rotterdam, The Netherlands; 9 Faculty of Pharmacy, University of Ljubljana, Ljubljana, Slovenia; 10 Chair and Department of Endocrinology, Jagiellonian University Medical College, Jakubowskiego str. 2, 30-688 Krakow, Poland; 11 University Hospital, Krakow, Poland; 12 Department of Clinical Biochemistry, Jagiellonian University Medical College, Krakow, Poland

**Keywords:** Medullary thyroid cancer, CCK2/gastrin receptor targeting, Molecular imaging, Therapy, Theranostics

## Abstract

**Introduction:**

Medullary thyroid cancer (MTC) is a rare malignant tumour of the parafollicular C-cells with an unpredictable clinical course and currently suboptimal diagnostic and therapeutic options, in particular in advanced disease. Overexpression of cholecystokinin-2 receptors (CCK2R) represents a promising avenue to diagnostic imaging and targeted therapy, ideally through a theranostic approach.

**Materials and methods:**

A translational study (GRAN-T-MTC) conducted through a Phase I multicentre clinical trial of the indium-111 labelled CP04 ([^111^In]In-CP04), a CCK2R-seeking ligand was initiated with the goal of developing a theranostic compound. Patients with proven advanced/metastatic MTC or short calcitonin doubling time were enrolled. A two-step concept was developed through the use of low- and high-peptide mass (10 and 50 μg, respectively) for safety assessment, with the higher peptide mass considered appropriate for therapeutic application. Gelofusine was co-infused in a randomized fashion in the second step for the evaluation of potential reduction of the absorbed dose to the kidneys. Imaging for the purpose of biodistribution, dosimetry evaluation, and diagnostic assessment were performed as well as pre-, peri-, and postprocedural clinical and biochemical assessment.

**Results:**

Sixteen patients were enrolled. No serious adverse events after application of the compound at both peptide amounts were witnessed; transient tachycardia and flushing were observed in two patients. No changes in biochemistry and clinical status were observed on follow-up. Preliminary dosimetry assessment revealed the highest dose to urinary bladder, followed by the kidneys and stomach wall. The effective dose for 200 MBq of [^111^In]In-CP04 was estimated at 7±3 mSv and 7±1 mSv for 10 μg and 50 μg CP04, respectively. Administration of Gelofusine reduced the dose to the kidneys by 53%, resulting in the organ absorbed dose of 0.044±0.019 mSv/MBq. Projected absorbed dose to the kidneys with the use of [^177^Lu]Lu-CP04 was estimated at 0.9±0.4 Gy/7.4 GBq. [^111^In]In-CP04 scintigraphy was positive in 13 patients (detection rate of 81%) with superior diagnostic performance over conventional imaging.

**Conclusion:**

In the present study, [^111^In]In-CP04 was shown to be a safe and effective radiopharmaceutical with promising theranostic characteristics for patients with advanced MTC.

## Introduction

Medullary thyroid cancer (MTC) is a rare malignant tumour of the parafollicular C-cells. A noticeable increase in the incidence of MTC is currently observed; although it constitutes only about 5% of thyroid malignancies, it is responsible for approximately 13% of all thyroid cancer-related deaths [1]. The clinical course of the disease ranges from indolent to aggressive, with early regional and distant spread. The 10-year relative survival rate in localized disease is excellent (above 95%), but drops significantly in patients with distant metastases (around 40%) [2].

While surgery consisting of total thyroidectomy and central or modified radical neck dissection is performed in patients without extranodal and/or distant disease with a curative intent, up to 50% of patients experience disease relapse with the development of regional or distant metastases or are already diagnosed initially with advanced disease [3]. Disease relapse is typically heralded by the increase in tumour markers calcitonin (Ct) and carcinoembryonic antigen (CEA). Several imaging modalities, including nuclear medicine imaging [4], are used to localize MTC lesions, overall with moderate detection rate. With advanced disease, systemic approaches including targeted therapy, chemotherapy and recently also peptide receptor radionuclide therapy (PRRT) are employed, again with limited efficacy [5]. An obvious need therefore exists to develop alternative diagnostic and therapeutic—preferably theranostic—strategies to control tumour growth.

With the overexpression of cholecystokinin-2 receptors (CCK2R)/gastrin receptors on the membrane of MTC cells, novel radiopharmaceuticals with high affinity to these receptors represent a potential effective tool for molecular diagnostics and therapy. A variety of CCK2 receptor-binding peptides were studied in the last three decades as ligands for radionuclide imaging and targeted therapy [6]; among those, one derivative—CP04 (DOTA-(DGlu)_6_-Ala-Tyr-Gly-Trp-Met-Asp-Phe-NH_2_)—showed highly promising characteristics for clinical translation [7, 8].

In this report, the final clinical results of the translational study GRAN-T-MTC are presented — a Phase I multicentre clinical trial of the indium-111 (^111^In) labelled CP04 ([^111^In]In-CP04), a CCK2 receptor (CCK2R)-seeking ligand, conducted within the ERA-NET framework on Translational Cancer Research (TRANSCAN) First Joint Transnational Call for Proposals 2011 (JTC 2011; “Validation of biomarkers for personalised cancer medicine”). The objectives of the GRAN-T-MTC study were to assess the safety of the intravenous administration of the CCK2R/gastrin analogue CP04, as well as the diagnostic potential, biodistribution, and dosimetry of [^111^In]In-CP04 in cancerous and normal tissue. The assessment also included the administration of the higher peptide mass amount better representing those during PRRT and the application of nephroprotection in order to acquire first solid patient data on the applicability of [^111^In]In/[^177^Lu]Lu-CP04 in the theranostic management of advanced MTC.

## Materials and methods

### Study approval and registration

This was a Phase I multicentre randomized, open, parallel-arm clinical trial with the investigational medicinal product (IMP) [^111^In]In-CP04 ([^111^In]In-DOTA-(DGlu)6-Ala-Tyr-Gly-Trp-Met-Asp-Phe-NH2), registered at www.clinicaltrials.gov (NCT03246659) and EudraCT (2015-000805-38). The trial was approved by the ethics committees in all three centres participating in the clinical part of the project. The Investigator’s Brochure (IB) and the Investigational Medicinal Product Dossier (IMPD) were submitted and approved by the national authorities of all participating centres.

### Study population

Patients meeting the inclusion criteria (Table 1) were enrolled after providing a signed informed consent for trial participation. Women of childbearing age and fertile men were asked to apply effective methods of contraception before and after enrolment throughout the study up to the last follow-up visit. If applicable, a pregnancy test was performed at the screening visit before enrolment in the study, on the day of radiopharmaceutical administration(s) and at the last follow-up visit. The assessment of baseline laboratory (complete blood count, biochemistry) and tumour-related signs and symptoms was performed before the start of the clinical trial, with appropriate follow-up.

**Table 1:** Inclusion and exclusion criteria for the clinical trial

Inclusion criteria	Exclusion criteria
Histologically documented medullary cancer of the thyroid	Patients with surgically treatable MTC
Presence of more than one distant or nodal, surgically untreatable metastases confirmed with either [^18^F]FDG PET-CT or contrast-enhanced CT (ceCT) or MRI or Doubling time (DT) of serum calcitonin concentration less than two years prior to study entry and negative imaging	Patients with history of second malignancy other than basal cell carcinoma of the skin
Karnofsky performance status > 50%	Participation in any other investigational trial within 3 months of study entry
Life expectancy of more than 6 months	Previous external beam radiation therapy within two years
Male or female patients aged >18 years without upper age limit	Organ allograft requiring immunosuppressive therapy
Ability to understand and willingness to sign a written informed consent document	Pregnancy, breast feeding
	Known hypersensitivity to gastrin analogues
	Patients with concurrent illnesses that might preclude study completion or interfere with study results
	Patients with bladder outflow obstruction or unmanageable urinary incontinence
	Clinical diagnosis of disseminated intravascular coagulation
	Serum creatinine >170 mmol/L, GFR < 40 mL/min
	Known history of hypersensitivity to Gelofusine or any other contraindications to Gelofusine infusion

### Study objectives and endpoints

The primary objective of the trial was the evaluation of the safety of the administration of [^111^In]In-CP04 at low mass amount of 10 μg CP04 and at high mass amount of 50 μg CP04 radiolabelled with 200±10% MBq of ^111^In. Therefore, the definition of the type, frequency, severity, timing, and relation to the studied radiopharmaceutical administration of Adverse Events assessed by CTCAE v4.0 was used as primary endpoint to define the safety and tolerability of [^111^In]In-CP04.

The secondary objectives of the study include the evaluation of dosimetry and biodistribution of the IMP with the corresponding endpoints being the determination of the (i) kinetic curve and biodistribution profile by assessment of [^111^In]In-CP04 uptake in normal organs, tumour, and metastases; (ii) effective dose equivalent (mSv) and absorbed doses (mGy) of normal organs; (iii) effect of the IMP administered mass amount on the biodistribution and nephroprotection (detailed in the “Study design” section); (iv) pharmacokinetics of [^111^In]In-CP04, metabolism and excretion based on blood and urine sampling analysis. Furthermore, the detection rate of [^111^In]In-CP04 scintigraphy compared to other diagnostic tests ( [^18^F]FDG PET/CT, ceCT, or MRI imaging) was used as endpoint to determine the diagnostic performance of [^111^In]In-CP04 scan.

### Study design

**Fig. 1:**
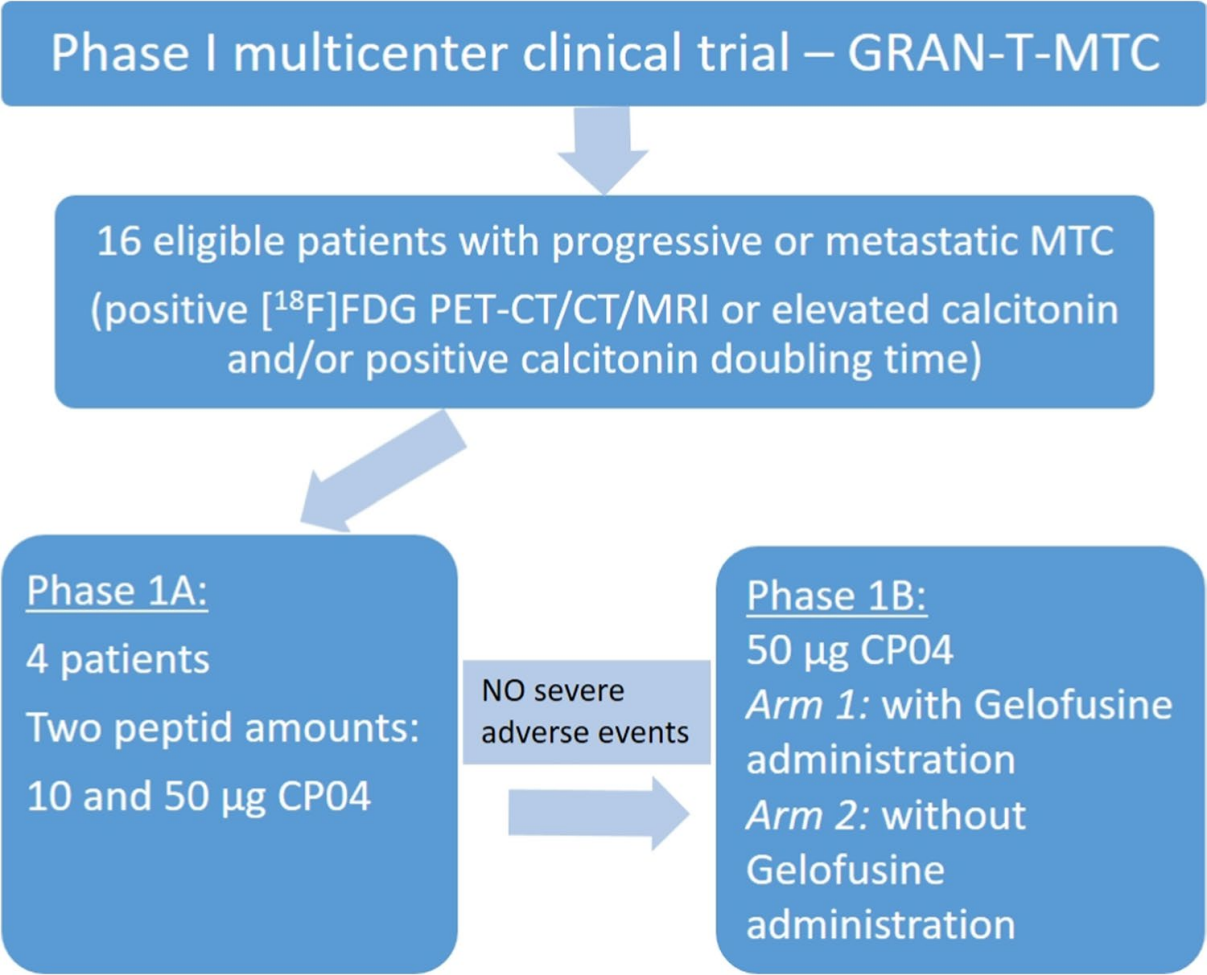
GRAN-T-MTC Trial design.

The clinical trial design comprised of two phases, 1A and 1B. In Phase 1A, the first 4 enrolled patients (3 patients with low-burden advanced disease and one with negative imaging and elevated Ct) were administered with the low mass amount of 10 μg [^111^In]In-CP04 as a safety step. If this dose was well tolerated, the high mass amount of 50 μg of [^111^In]In-CP04 was injected after 2 weeks. The mass amount of 50 μg was assumed to be the potential therapeutic amount if the compound was to be labelled with the β-emitter lutetium-177 (^177^Lu). According to the study protocol, subsequent tracer administrations were performed at intervals of at least 2 weeks, providing that no serious adverse events (SEA) were observed (Fig. 1) [9]. After safety confirmation, in Phase 1B only 50 μg of [^111^In]In-CP04 was administered to all further enrolled patients. Additionally, these patients were randomly assigned into Arm 1 with Gelofusine and into Arm 2 without Gelofusine co-administration, respectively.

Detailed physical examinations with vital sign assessment, 12-lead ECG, and laboratory testing were performed to investigate the safety of intravenous administration of [^111^In]In-CP04. The overall safety profile of the tested CCK-2/gastrin receptor ligand as characterized by timing, type, frequency, severity of adverse events (AE), and laboratory abnormalities was based on Common Terminology Criteria for Adverse Events (CTCAE) version 4.0. Since this was a Phase I clinical trial, the project was assumed as having a high-risk potential for the patient, and therefore, special care was taken with the monitoring of the study. In total, eight control visits were carried out over 4 months of the follow-up with physical examination, laboratory testing, and ECG. Additionally, Ct and proCt concentrations were measured after each [^111^In]In-CP04 injection at predefined time points (0, 2, 5, 10, 20 min).

### [^111^In]In-CP04 preparation

Within the preclinical phase of GRAN-T-MTC, [^111^In]In-CP04 was evaluated for pharmaceutical characteristics and validated in animal models. A clinically useful kit formulation for CP04 radiolabelling with ^111^In for use in the clinical phase has been developed [10]. The results of the preclinical part of the project, as well as the protocol of the clinical study, have been already published elsewhere [9, 11].

The cold CP04 kits with mass amounts of 10 and 50 μg for on-site radiolabelling were used. Radiolabelling with ^111^In (Indium chloride, Mallinckrodt Medical, Petten) was performed at 90°C using a standardized protocol resulting in a final preparation containing 200±10% MBq [^111^In]In-CP04 ready for injection [11]. The radiolabelling procedure was performed in an environment suitable for handling radioactive material at the Radiopharmacy Department or at the site specially designated by the Nuclear Medicine Department in each participating centre. Quality control by high-performance liquid chromatography (HPLC) ensured a radiochemical purity of [^111^In]In-CP04 of >95% for all applications.

Prior to any patient administration, the Standard Operating Procedures (SOP) were developed for radiolabelling and quality control procedures, and the personnel of each clinical centre was trained and qualified on these SOP for their implementation in accordance with the IMPD.

### Gamma camera calibration and validation for quantitative SPECT imaging

Prior to the start of the clinical trial, a cross-calibration of all SPECT/CT cameras and the dose calibrators in the participating centres was performed using a Jaszczak SPECT phantom to ensure the accuracy and reproducibility of calculations. An empty Jaszczak phantom with all inserts removed was filled with a homogenous ^111^In solution (50 MBq). The acquisition and reconstruction protocol for the phantom were identical to the acquisition settings for the 24 h post-injection quantitative SPECT/CT scan conducted during the trial. These procedures were standardized across sites according to the study protocol and were carried out under supervision of an experienced study dosimetrist or physicist.

### Radiopharmaceutical administration and imaging

The duration of [^111^In]In-CP04 administration with the low and high mass amount was 5 min and 30 min, respectively. During the injection of the IMP, a dynamic scan of the kidneys and stomach was performed for 30 min (anterior and posterior acquisition). The imaging protocol included a mandatory whole-body scan (WBS) at 1 h, 4 h, 24 h, and 48 h p.i. (table shift speed 12 cm/min). A quantitative SPECT/CT scan of the abdomen was performed at 24 h p.i. as well as an additional non-quantitative SPECT/CT scan of the area where pathological lesions were detected during WBS, if the scan duration was tolerated by the patient. Gelofusine (B. Braun, Melsungen, Germany) was administered as an infusion of 1 mL/kg body weight over 10 min followed by a 3-h infusion of 0.02 mL/kg/min after the injection of the IMPD. Physiological parameters (heart rate, blood pressure, oxygen saturation) were repeatedly measured during and after the [^111^In]In-CP04 infusion as per study protocol.

### Biodistribution and dosimetry evaluation

During the [^111^In]In-CP04 administration blood samples for dosimetry and pharmacokinetic purposes were taken at the set time intervals according to the study protocol: biodistribution and dosimetry data were acquired based on serial planar and SPECT/CT images, and blood and urine collection at regular time intervals. Urine samples were collected up to 24 h after the tracer injection to determine the bladder wall absorbed dose. On the WBS planar imaging at 1, 4, 24, and 48 h (including standard activity) and quantitative SPECT/CT imaging at 4 and 24 h, the regions of interest (ROI) were drawn over the kidneys and over the lesions when visible. The counts in the ROIs were quantified to activity by cross-calibration with the activity determined in the corresponding volumes of interest (VOIs) drawn on the quantitative SPECT/CT image at 24 h. Time-activity curves were determined by fitting exponential curves through the uptake data. Time integrated activity coefficients (TIAC) were calculated for ^111^In and ^177^Lu by integration of the TIACs multiplied with their physical decaty functions. The absorbed dose per unit activity in each organ was determined by multiplication with the ^111^In and ^177^Lu S-values according to the MIRD formalism. The blood time-activity curve was used to estimate the absorbed dose to the bone marrow. The activity concentration in the bone marrow was considered to be equal to the blood activity concentration. The activity and volumes of the urine samples were used to determine the urinary clearance rate, and with the dynamic bladder model, the TIAC in the bladder and the absorbed dose to the bladder wall were determined. The effect on the kidney absorbed dose after co-administration of the nephroprotective agent Gelofusine was evaluated. All dosimetry calculations were performed with the Hermes-OLINDA v2.1 hybrid 2D dosimetry software.

### Ct and proCt measurements

During the [^111^In]In-CP04 administration blood samples for Ct and proCt concentrations measurements were taken at predefined time intervals according to the study protocol. The measurements were performed at the Central Laboratory participating in the external international quality assessment scheme, served by RANDOX Laboratories Ltd., Crumlin, UK. The enzyme-linked immunosorbent assay (ELISA) with the Calcitonin EIA-3648 and Procalcitonin (Human) EIA-5291reagent kits (DRG®Instruments GmbH,Germany) and ELx808 spectrophotometer (BioTek R Instruments, Inc., Winooski, Vermont, United States) was used. The obtained blood samples were centrifuged (15 min, 1000 g, 18°C) and the serum after separation from the clot was stored at −80°C locally at each centre. Serum samples were sent frozen in dry ice to the Central Laboratory. For each shipment, a temperature data logger was used to monitor and record temperature values during the whole period.

### Assessment of the diagnostic potential of [^111^In]In-CP04

For the evaluation of the acquired images, qualitative visual analysis was used to determine the presence/absence and the number of lesions identified by scintigraphy based on abnormal tracer uptake (defined as clearly discernible uptake above background concentration—regional blood pool). The number and identity of the lesions with increased abnormal uptake detected per verifiable organ or body region relative to those detected by conventional imaging were assessed. Identification of the time points p.i. with the highest observed number of lesions and the highest tumour/background ratios for both 10 and 50 μg mass amounts was performed to evaluate the influence of mass amount on tumour and normal organ uptake. In all clinical centres, the scintigraphy scans were evaluated by two experienced nuclear medicine specialists.

### Statistical analysis

The assessed variables were analyzed and shown descriptively. *T-*test (paired/unpaired, as appropriate) was used for comparison of variables. All statistical calculations were performed using SPSS and STATA software packages.

## Results

### Patient characteristics

Sixteen patients with histologically proven and advanced MTC were enrolled in the study based on the specific inclusion and exclusion criteria. [^18^F]FDG PET/CT, ceCT, or MRI imaging (initiated at Ct concentrations above 150 pg/mL or clinical suspicion of disease at a specific site) was positive in 12 patients; in four, short calcitonin doubling time (CtDT) with negative conventional imaging (CT of the neck/thorax, abdomen; [^18^F]FDG PET/CT) was present. All patients included in the study had a baseline Karnofsky performance status >50%. The baseline laboratory results and characteristics of the study group are presented in Tables 2 and 3.

**Table 2:** Baseline laboratory test results

Blood test	Units (SI)	Reference range	Median	Q1	Q3
Hemoglobin	g/L	110–170	145	136	161
Hematocrit	L/L	0.35–0.5	0.44	0.41	0.49
WBC	10^9/L	3–10	6.37	5.25	8.1
PLT	10^9/L	125–400	260	223	332
TSH	mIU/L	0.2–4.78	0.61	0.15	3
FT3	pmol/L	3.1–6.8	4.34	3.84	5.2
FT4	pmol/L	10.3–25	20.2	18.6	23.7
Sodium	mmol/L	136–145	140	142	142
Potassium	mmol/L	3.5–5.5	4.24	4.19	4.5
Calcium	mmol/L	2.1–2.65	2.26	2.24	2.34
Creatinine	μmol/L	55–115	63	56	66
Albumin	g/L	32–55	44	43	46,5
Bilirubin	μmol/L	0–21	8.57	7.7	10.1
AST	nkatal/L	up to 583	346.7	240	350.07
ALT	nkatal/L	up to 750	350	266	533
GFR	mL/s/1.73^2	>60	90	60	90
Ct	pg/mL	<13 (F); <30 (M)	1520.5	330	27212
proCt	pg/mL	<50.0	205.7	1.13	194.7
CEA	ng/mL	0.0–5.20	23.1	7.05	299.3

*Q1 and Q3*, first and third quartile; *F*, female; *M*, male

**Table 3:** Patient characteristics

Gender	11 female, 5 male
Age	30-66 years
Age at diagnosis	11-63 years
Weight	45-118 kg
Genetic status	2 MEN2A 1 MEN2A/FMTC 2 MEN2B 10 sporadic 1 unknown
TNM stage at diagnosis	T1bN0M0-T4N2M1
TNM stage at presentation	TxN1M0-T4N2M1
Surgical procedures (No)	1-5
Comorbidities	3 pheochromocytoma^*^ 2 limb palsy 2 neprolithiasis 1 ectopic ACTH secretion^*^ 1 arterial hypertension 1 diabetes mellitus type 2 1 pleomorphic adenoma 1 Hashimoto thyroiditis 1 anemia

*MEN*, multiple endocrine neoplasia; *FMTC*, familial medullary thyroid cancer; *ACTH*, adrenocorticotropic hormone; ^*^recurrent/persistent/metastatic disease excluded by clinical presentation and biochemical and imaging evaluation

### Safety of the [^111^In]In-CP04 administration

**Fig. 2:**
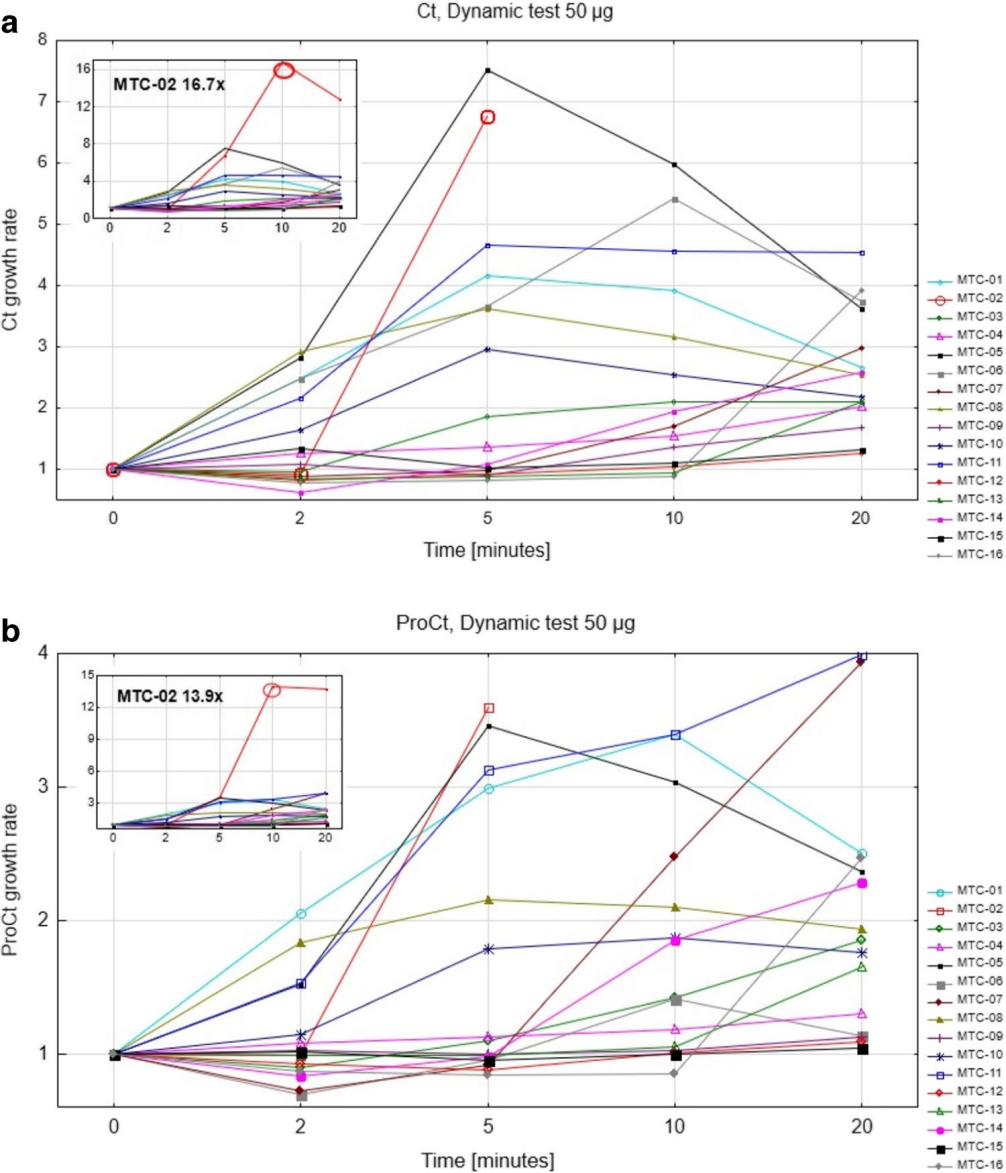
Calcitonin (**a**) and procalcitonin (**b**) growth rates during the dynamic test after injection of 50 μg CP04 in all patients (MTC01-MTC16), expressed as multiple of the baseline value

Patients were closely monitored for potential AE and the need of concomitant medications; no such events were observed throughout the clinical trial. During the injection of both 10 and 50 μg mass amounts, only two patients (MTC01, MTC13) suffered from transient tachycardia and mild, short-lasting flush. Since transient tachycardia (up to 128 beats/min lasting less than 5 minutes) in the patient MTC01 was already observed before, the relation between the injection of the IMP and the AE is not clear. There was a marked increase in Ct and proCt concentrations in the blood after tracer injection in several patients with large metastatic burden of MTC. Figure 2 represents the Ct and proCt results for the whole patient group after the CP04 injection (50 μg; the delay of the maximal growth rate of Ct and proCt resulted from the slow infusion of the radiolabelled peptide due to safety reasons). In three cases (MTC06, MTC10, and MTC12), the increase of Ct and proCt exceeded 100.000 pg/mL and 500.000 pg/mL, respectively; however, no symptoms were reported by the patients and no significant changes in the monitored physiological parameters were observed. In individual patients, the dynamics of Ct and proCt concentrations appeared to change in correlation.

The laboratory test results did not change significantly during the control visits. No significant changes in the ECG recordings were observed. The performance status assessed according to Karnofsky index did not change.

### Pharmacokinetics, biodistribution, and dosimetry

**Fig. 3:**
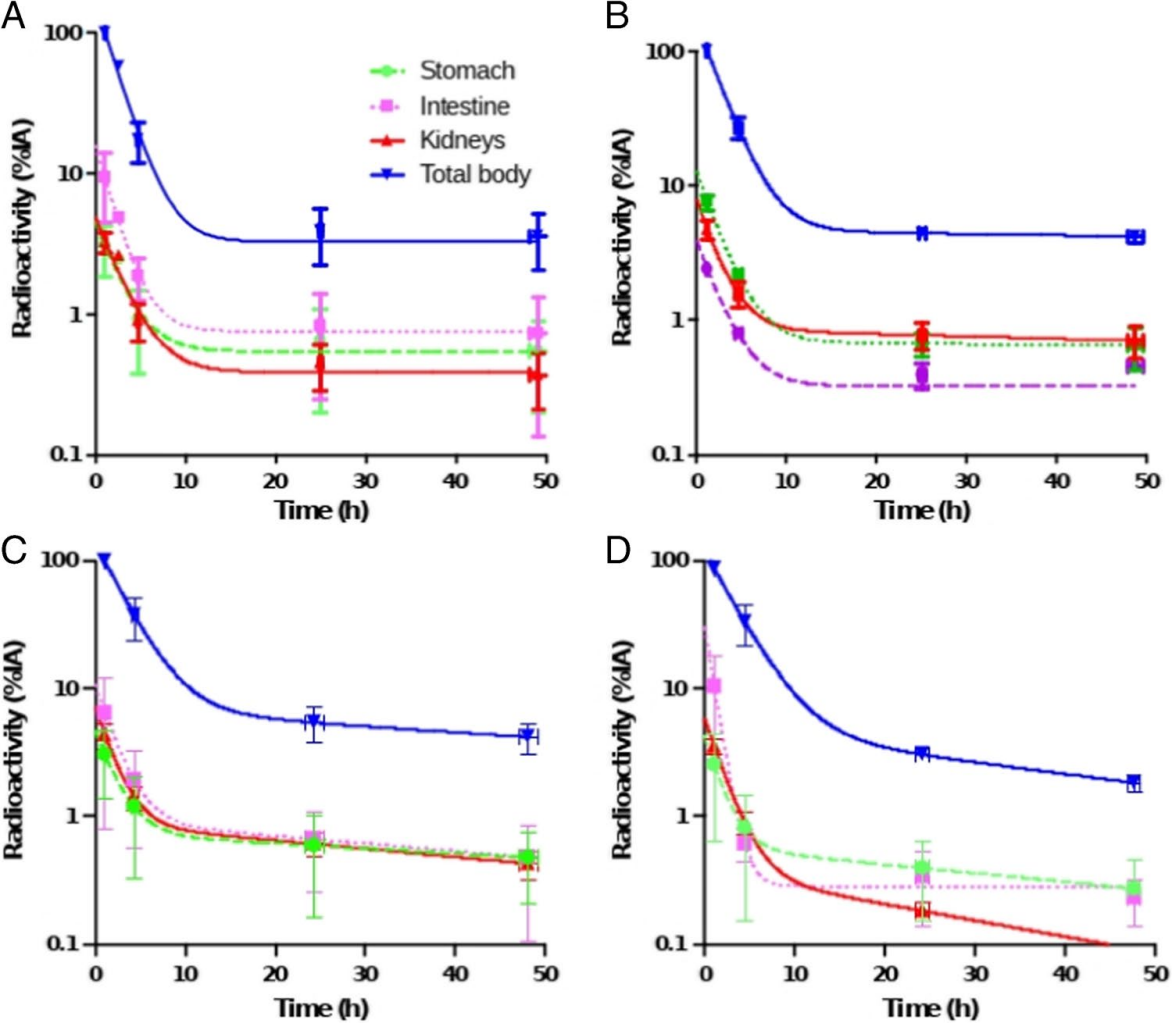
Pharmacokinetics of [^111^In]In-CP04. Comparison of [^111^In]In-CP04 pharmacokinetics of 10 μg (panel **A**) vs 50 μg (panel **B**) in patients MTC01-MTC04; comparison of [^111^In]In-CP04 pharmacokinetics of 50 μg without gelofusine (*N*=10, panel **C**) with gelofusine (*N*=6, panel **D**). The curves indicate exponential fits through the data

Blood sampling of [^111^In]In-CP04 was performed in patients who were administered with both 10 and 50 μg mass amounts. Rapid clearance from the blood was observed in both cases (Fig. 3).

**Fig. 4:**
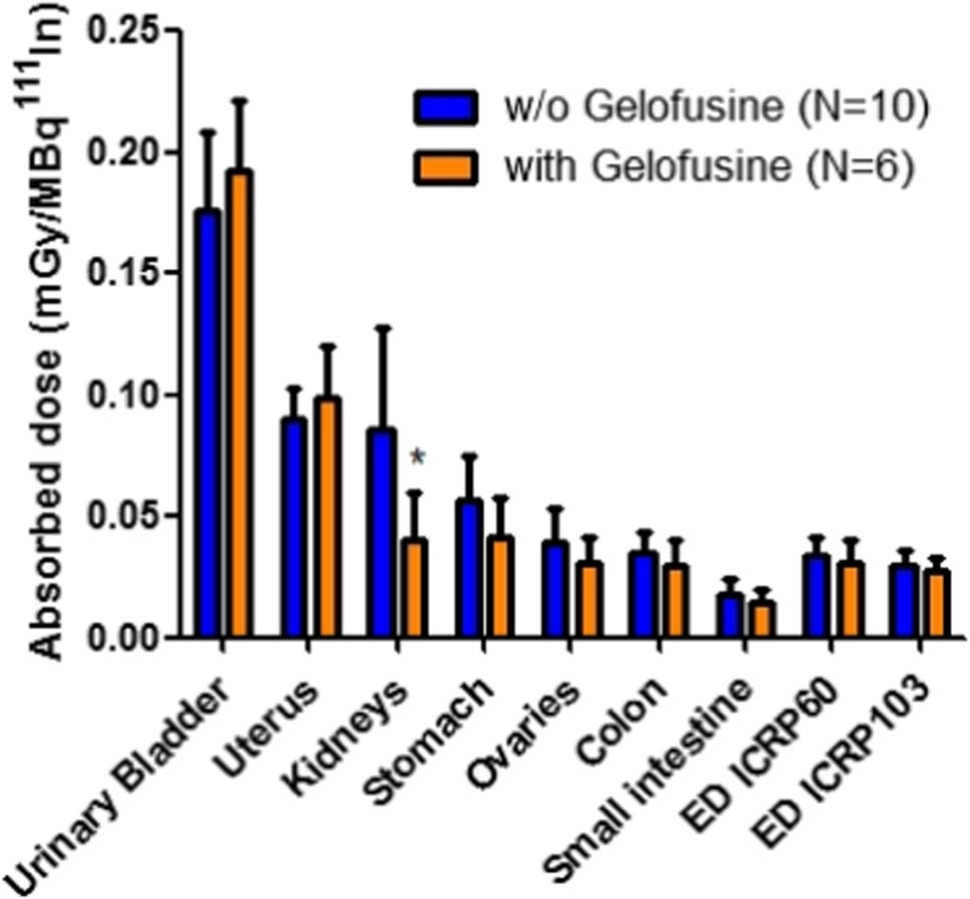
Dosimetry results of [^111^In]In-CP04 absorbed dose per injected activity (mGy/MBq). *Significant difference (*P*<0.01) in organ absorbed dose

The observed uptake in the intestines, stomach, and kidneys was not dependent on the mass amount. Preliminary dosimetry assessment revealed the highest dose to urinary bladder, followed by the kidneys and stomach wall. The effective dose for 200 MBq of [^111^In]In-CP04 was estimated at 7±3 mSv for 10 μg CP04 and 7±1 mSv for 50 μg CP04. Blood sampling in patients enrolled to Phase 1B revealed a bi-exponential blood clearance with an α-half-life of 12 min vs 34 min (w/o vs with Gelofusine) and a β-half-life of 114 min vs 262 min (w/o vs with Gelofusine). Administration of Gelofusine reduced the dose to the kidneys by 53%, resulting in the absorbed dose of 0.044±0.019 mSv/MBq (*P*<0.05); the absorbed dose to the stomach wall was lower than the kidney absorbed dose and was not affected by Gelofusine (Fig. 4). With theranostic application as the potential goal, the acquired data for 50 μg of [^111^In]In-CP04 were translated to estimate organ doses of [^177^Lu]Lu-CP04. Administration of Gelofusine resulted in a significant, 64% reduction in the absorbed dose to the kidney (0.9±0.4 Gy/7.4 GBq of [^177^Lu]Lu-CP04), with the absorbed dose to the stomach wall highly dependent on regional uptake. Summary of dosimetry results is shown in Table 4.

**Table 4:** Dosimetry results of the absorbed dose (mGy/MBq) for target organs after [^111^In]In-CP04 scintigraphy; evaluation of the effect of Gelofusine infusion

Patient	Kidneys			Stomach		
	10 μg	50 μg	50 μg + Gelofusine	10 μg	50 μg	50 μg + Gelofusine
MTC01	0.102	0.197	N/A	0.134	0.061	N/A
MTC02	0.077	0.146	N/A	0.053	0.047	N/A
MTC03	0.098	0.127	N/A	0.067	0.065	N/A
MTC04	0.052	0.058	N/A	0.056	0.051	N/A
mean** ± **SD	0.082±0.022	0.132±0.057		0.077±0.038	0.056±0.008	
*P*-value		0.087			0.299	
MTC05		0.121	N/A		0.095	N/A
MTC06		N/A	0.057		N/A	0.055
MTC07		0.082	N/A		0.100	N/A
MTC08		N/A	0.062		N/A	0.040
MTC09		0.074	N/A		0.105	N/A
MTC10		N/A	0.068		N/A	0.076
MTC11		0.096	N/A		0.092	N/A
MTC12		N/A	0.039		N/A	0.074
MTC13		0.043	N/A		0.049	N/A
MTC14		N/A	0.023		N/A	0.031
MTC15		0.042	N/A		0.038	N/A
MTC16		N/A	0.018		N/A	0.030
mean** ± **SD		0.099 ± 0.047	0.044 ± 0.019		0.070 ± 0.024	0.051 ± 0.019
***P*-**value			0.024			0.140

*N/A*, not available/applicable

### MTC targeting

**Fig. 5:**
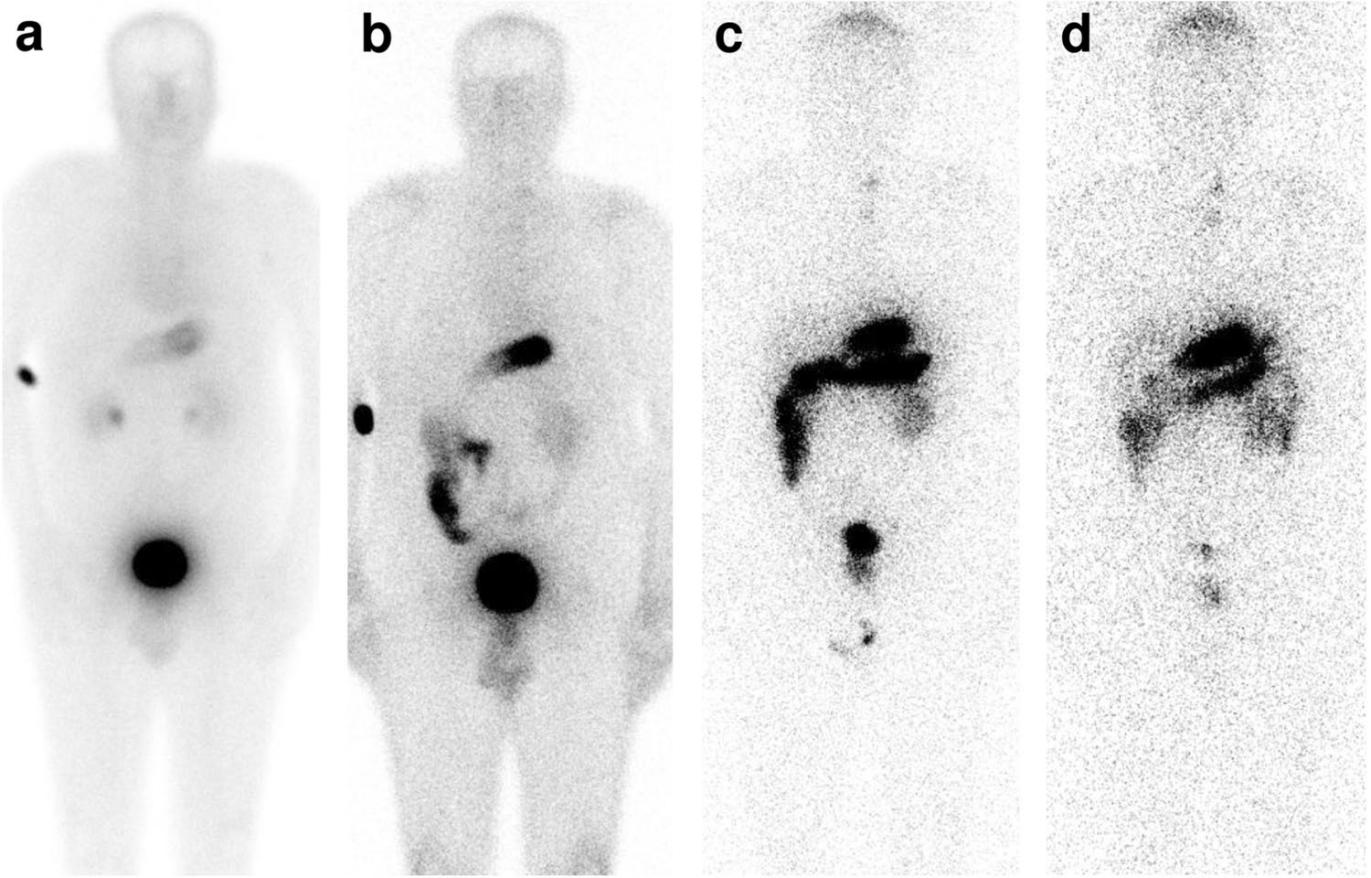
Whole-body dynamic distribution after intravenous administration of 50 μg of [^111^In]In-CP04 immediately after injection (**a**), at 1h (**b**), 24h (**c**), and 48h (**d**), patient MTC04

In the first four patients (MTC01-MTC04, Phase 1A), the quality of the images was comparable in terms of detected focal lesions as well as in the clinical assessment of the disease spread regardless of the amount of [^111^In]In-CP04; whole-body dynamic distribution of the radiopharmaceutical is shown in Fig. 5. Tracer uptake in seven small visible lesions was relatively low on WBS but quite high on SPECT/CT imaging (Table 5). No statistical differences were found for target/non-target (T/nT) ratios for most clearly visible lesions detected by 10 μg of [^111^In]In-CP04 for planar scans; for SPECT/CT imaging, T/nT was higher when 50 μg of labelled peptide was used.

**Table 5:** Target/non-target (T/nT) ratios obtained after 10/50 μg (Phase 1A) of [^111^In]In-CP04 injection for whole-body scan (WBS) and SPECT/CT

Mass amount	WBS		SPECT/CT
	24h p.i.	48h p.i.	24h p.i.
10 μg	2.1 (1.8–2.3)	2.0 (1.7–2.6)	12.1 (5.9–14.1)
50 μg	1.9 (1.1–2.5)	2.4 (1.8–2.7)	10.7 (4.8–21.0)
*P*-value	NS	NS	NS

*NS*, not significant. T/nT ratios shown as median (first–third quartile)

**Fig. 6:**
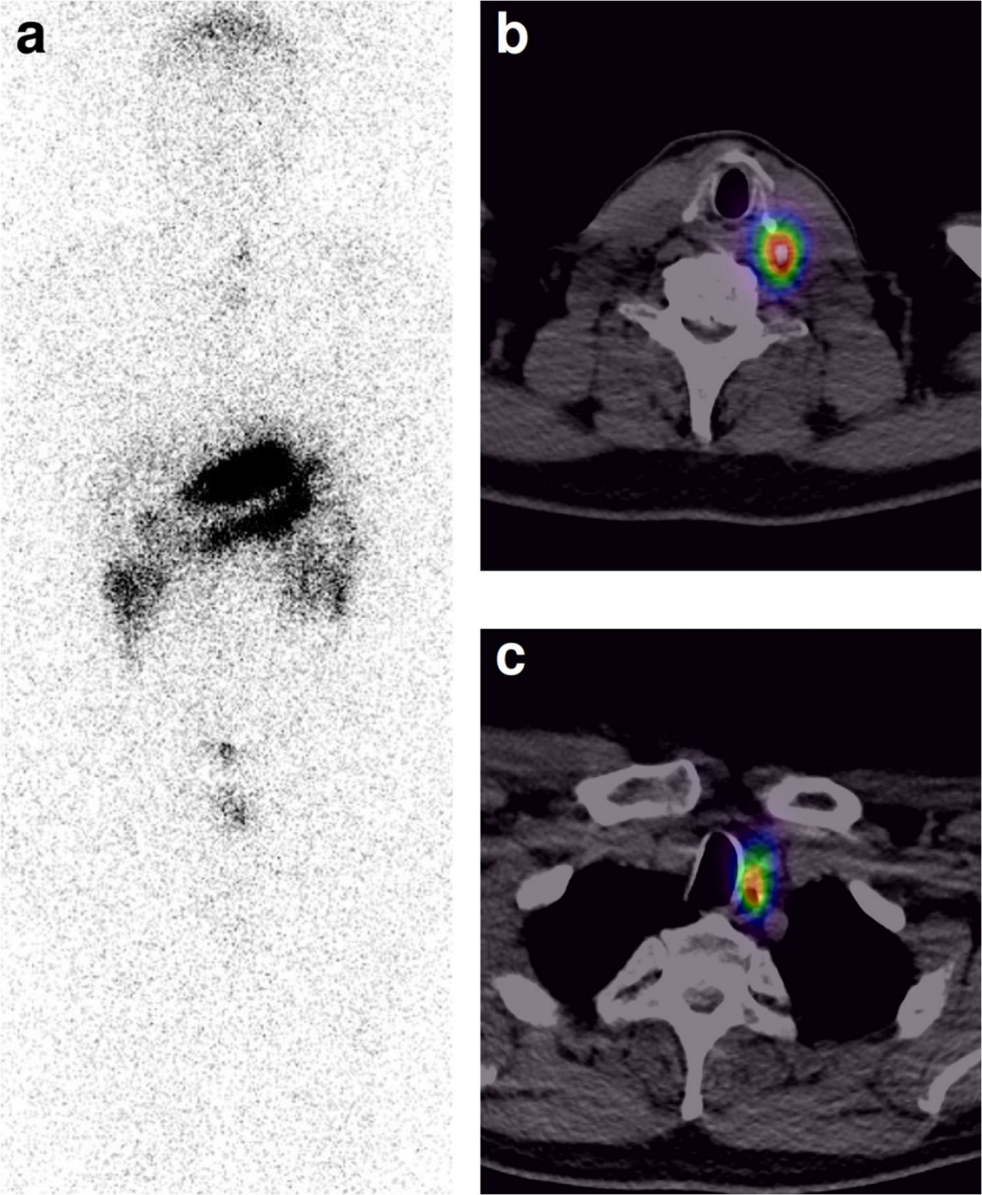
A 56-year old male (MTC04) with sporadic MTC, basal Ct concentration: 478 pg/mL, short calcitonin doubling time, no tumour tissue was detected by conventional imaging methods. Uptake of [^111^In]In-CP04 in two small left paratracheal lesions is visible both on planar (**a**) and SPECT/CT imaging (**b**, **c**). The patient underwent surgery of the upper resectable lesion; histopathology: lymph node metastasis of MTC

**Fig. 7:**
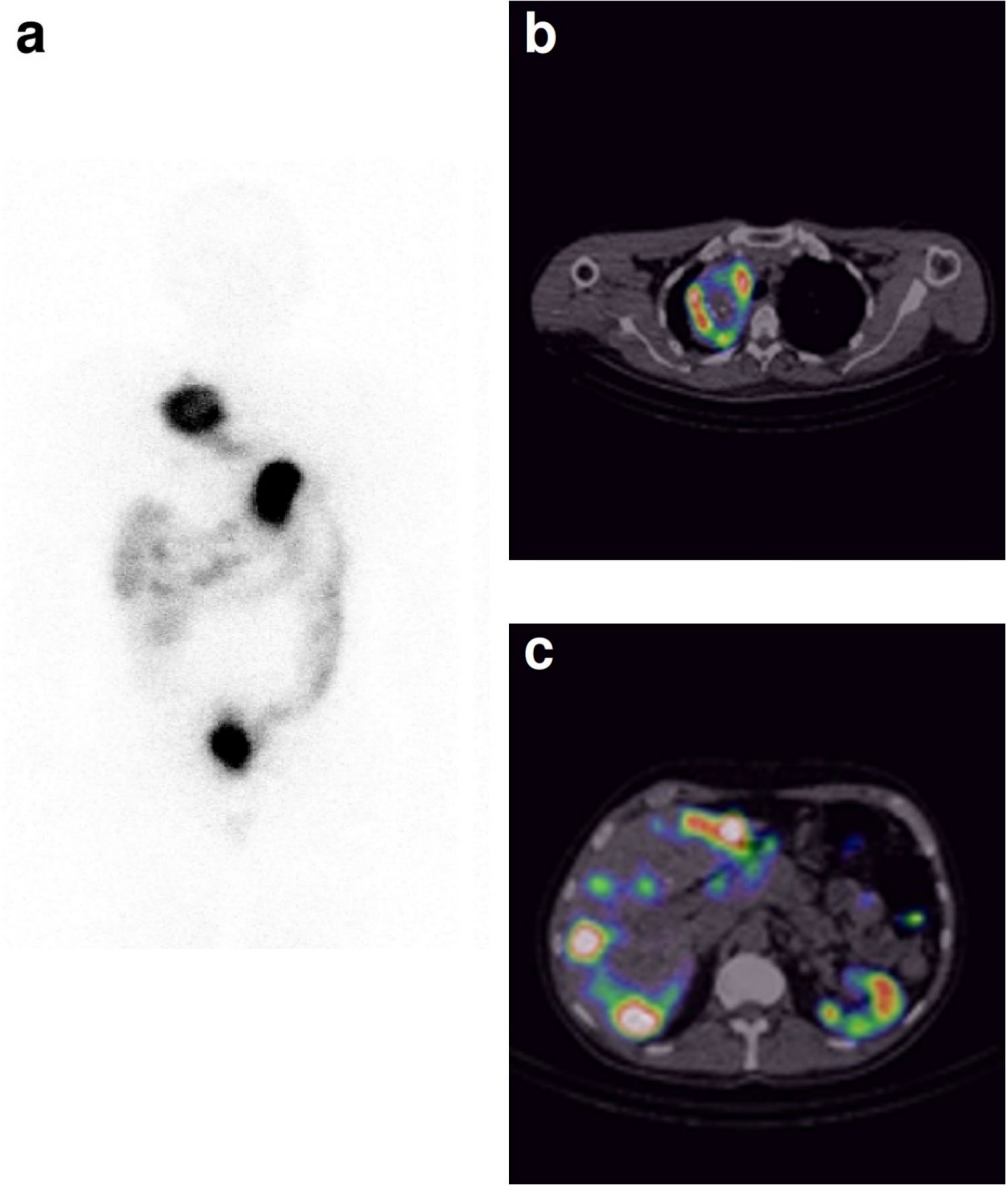
A 49-year old male (MTC10) with advanced sporadic MTC, basal Ct 1288 pg/mL. Uptake of [^111^In]In-CP04 in a large tumour mass in the mediastinum on the right (**b**) and numerous liver metastases (**c**) is visible with considerable detail on SPECT/CT in comparison to planar (**a**) imaging

**Fig. 8:**
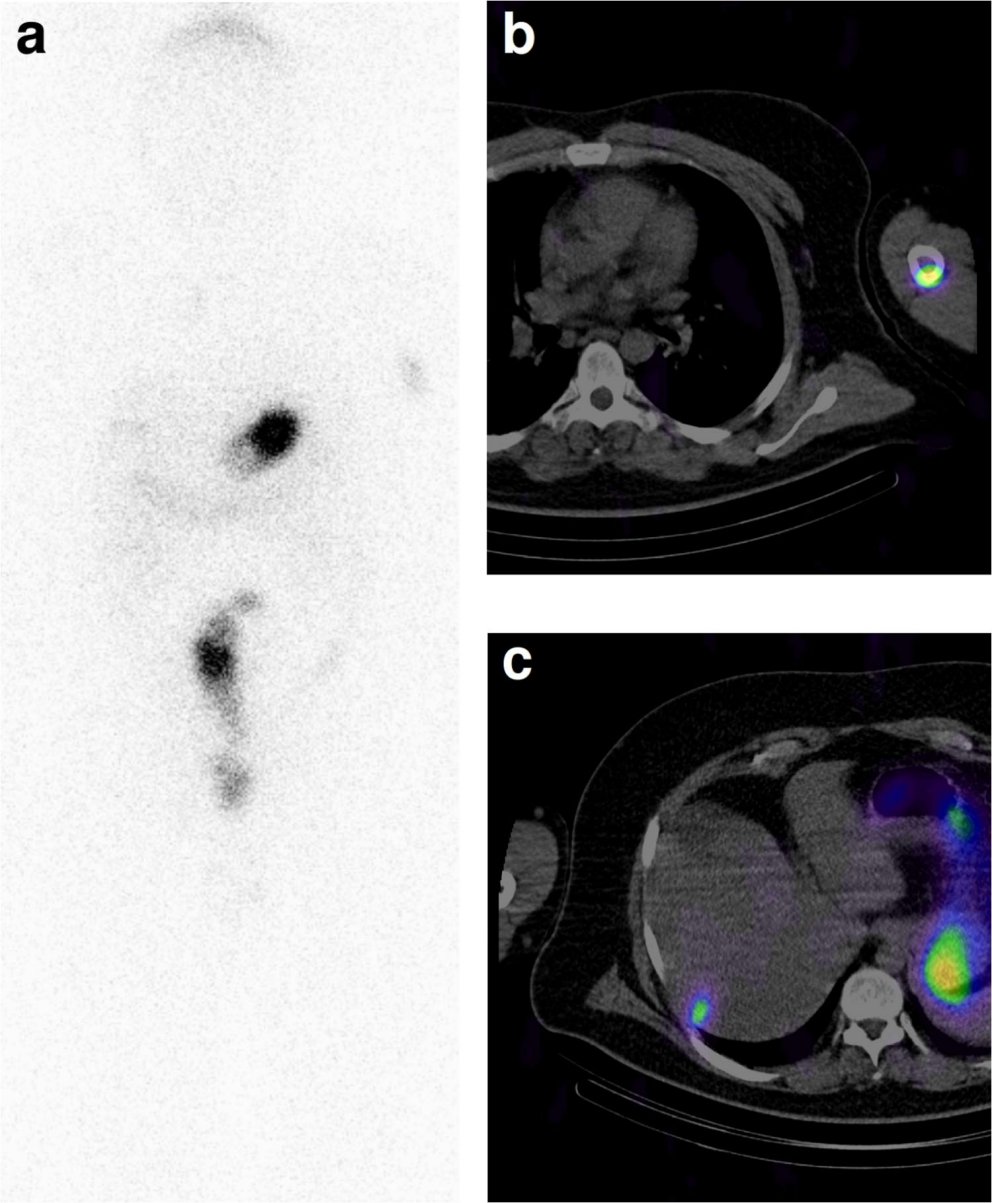
A 42-year old male (MTC16) with advanced MTC in MEN2a, basal Ct 95 pg/mL. Uptake of [^111^In]In-CP04 in a large left humeral metastasis (**b**) and in a small, previously undetected liver metastasis (**c**). On the whole-body distribution (**a**), some metastatic mediastinal lymph nodes are also visible

In the enrolled patients, the uptake of [^111^In]In-CP04 in MTC lesions was mainly in the neck and mediastinal regions (Fig. 6). In the advanced cases, metastases in the liver and other organs and tissues were visible (Figs. 7 and 8). Two patients (MTC01 and MTC04, Phase 1A) underwent surgery based on scintigraphy results and the largest lesions located on the neck were removed. Histopathology examination confirmed metastatic MTC in both patients. In patient MTC04 (Fig. 5), two lesions were detected by the [^111^In]In-CP04 imaging which were negative on CT and MRI; only one metastasis was surgically accessible. In one patient (MTC03), all imaging examinations were inconclusive which did not allow for surgical treatment. In another patient (MTC11, Phase 1B) with negative [^111^In]In-CP04 scan, the two small lesions in the left thyroid bed suspicious for MTC recurrence based on contrast-enhanced MRI were also negative in histopathology. In patient MTC14, MRI detected more liver lesions than [^111^In]In-CP04. Overall, in patient-based analysis, [^111^In]In-CP04 was negative in three patients (two with negative conventional imaging; one with histologically proven false-positive liver lesion on MRI) and positive in 13 patients (detection rate of 81%), while conventional imaging combined was negative in four patients (Table 6). In lesion-based analysis, [^111^In]In-CP04 demonstrated superior diagnostic performance over conventional imaging (including false-positive MRI finding in patient MTC11) in six patients with more detected lesions; in two patients, the findings of [^111^In]In-CP04 were complementary to conventional imaging.

**Table 6:** Results summary of [^111^In]In-CP04 scintigraphy in relation to the reference/initial imaging method

Patient	Conventional imaging (modality)	[^111^In]In-CP04	Performance of [^111^In]In-CP04
MTC01	Negative (CT, FDG PET/CT)	Cervical LNN	Superior
MTC02	Cervical LNN (MRI)	Cervical LNN	Equivalent
MTC03	Negative (MR)	Negative	Equivalent
MTC04	Cervical LNN (US) negative (CT, FDG PET/CT)	Cervical LNN mediastinal LNN	Superior
MTC05	Mediastinal LNN (SSTR, FDOPA PET/CT) negative (FDG PET/CT)	Mediastinal LNN	Equivalent
MTC06	Cervical LNN (CT) mediastinal LNN (CT) liver lesions (CT)	Cervical LNN mediastinal LNN liver lesions	Equivalent
MTC07	Mediastinal LNN (CT)	Cervical LNN	Equivalent/complementary
MTC08	Negative (CT)	Negative	Equivalent
MTC09	mediastinal LNN (CT, FDG PET/CT)	Mediastinal LNN	Equivalent
MTC10	Mediastinal LNN (FDG PET/CT) hilar LNN (FDG PET/CT)	Mediastinal LNN hilar LNN liver lesions	Superior
MTC11	Negative (CT) liver lesions (MRI)	Negative	Superior (histologically confirmed false-positive liver lesion)
MTC12	Cervical LNN (CT) mediastinal LNN (CT) liver lesions (CT)	Cervical LNN mediastinal LNN liver lesions	Equivalent/complementary
MTC13	Cervical LNN? (CT) mediastinal LNN? (CT) liver lesions (CT) skeletal lesions (CT)	Cervical LNN mediastinal LNN liver lesions skeletal lesions	Superior
MTC14	Liver lesions (CT, MRI)	Liver lesions	Equivalent (more lesions detected on MRI)
MTC15	Mediastinal LNN (CT) skeletal lesions? (MRI)	Mediastinal LNN hilar LNN	Superior (likely hemangiomas on MRI, no uptake in skeletal lesions)
MTC16	Mediastinal LNN (FDG PET/CT) liver lesions (FDG PET/CT) skeletal lesions (FDG PET/CT)	Mediastinal LNN liver lesions skeletal lesions	Equivalent

*LNN*, lymph nodes; ?, suspected

## Discussion

The GRAN-T-MTC study represents a joint effort of the GRAN-T-MTC consortium to develop a diagnostic and potentially therapeutic radiopharmaceutical for imaging of MTC. The results in the present study suggest that the safety profile of the new radiopharmaceutical [^111^In]In-CP04 is appropriate for clinical application and diagnostic performance suitable for clinical use. Moreover, projected absorbed doses to critical organs if substituting ^111^In with a beta emitting radionuclide ^177^Lu suggest that repeated therapeutic applications of the radiopharmaceutical would be safe.

In the setting of the first-in-human application of the IMP, evaluation of the safety of the compound was the primary goal. Due to the concern of the potential side effects triggered by the release of Ct by the radiopharmaceutical (analogous to the currently unavailable Pentagastrin® test), Ct concentrations were monitored during and after the application of [^111^In]In-CP04. There is accumulating evidence in the literature on proCt as an alternative biomarker to Ct. The growing interest in the biomarker is based on the robustness of the available assays and stability of the samples in comparison to established Ct measurements [12], although the appropriate cut-off values are still debated. The effectiveness of the biomarker was demonstrated both in the setting of primary diagnosis as well as disease recurrence [13, 14]. In the present work, proCt was investigated in parallel with Ct. Overall, no significant AE related to the IMP were observed, regardless of the mass amount administered (10 and 50 μg). In addition, patients with large absolute or relative increase of Ct and proCt also suffered no AE; both biomarkers demonstrated comparable dynamics in individual patients (Figs. 1 and 2), confirming recent reports using penatgastrin stimulation [15, 16]. Application of [^111^In]In-CP04 was therefore considered safe, in keeping with the previous and recent studies using similar compounds [6, 17]; according to the results, proCt may also be considered an alternative biomarker to Ct, as already suggested by the above-mentioned studies and analyses.

Pharmacokinetics of [^111^In]In-CP04 were overall favourable with rapid clearance from blood and almost exclusive renal elimination. After 24 h, only the intestines, stomach, and kidneys revealed limited retention of activity, however on a low level with <1% of IA, resulting in an overall lower radiation dose than predicted in preclinical studies [11] with about 7 mSv for 200 MBq.

In comparison to conventional imaging modalities, [^111^In]In-CP04 demonstrated overall higher detection rate for metastatic lesions. It is well accepted that the molecular imaging modalities outperform conventional imaging (US, CT, and MRI in the present study) in the setting of recurrent disease [18], in particular PET/CT imaging with [^18^F]F-DOPA [19–21]. However, in the present study, most commonly used molecular imaging method for restaging prior to enrolment was diagnostically inferior [^18^F]FDG PET/CT [20, 22] (only positive in three patients in the present series with typically high CEA concentrations from 80 to 2087 ng/mL) due to the limited availability of [^18^F]F-DOPA; somatostatin receptor imaging was used in several patients, but its limited diagnostic performance in comparison to [^18^F]F-DOPA is also well described [20, 23]. With patient-based detection rate of over 80%, [^111^In]In-CP04 slightly outperformed conventional and molecular imaging modalities (detection rate of 75%) in the present study. These results are comparatively high as according to recent meta-analyses, even [^18^F]F-DOPA as a molecular imaging method of choice had a reported pooled patient-based detection rate of 66% [24]. However, the patient population in our study was highly preselected and small, which likely affected the results; moreover, the reported detection rate in the mentioned meta-analysis was likely affected by the relatively low Ct concentrations (above 150 pg/mL in only 73% of patients). In lesion-based analysis, additional sites of disease were detected in two patients and in two more, the findings were complementary to conventional imaging, as seen in patient MTC14, where a higher number of liver metastases was detected on MRI in comparison to [^111^In]In-CP04, which is likely related to the size of the lesions—a limitation already described for [^18^F]F-DOPA in comparison to MRI [25]. With routine whole-body SPECT/CT imaging performed instead of planar imaging and optional focused SPECT/CT acquisition of the suspected target lesions, the diagnostic performance would likely additionally improve, but the imaging protocol required for dosimetry assessment precluded the routine use of the whole-body hybrid imaging approach due to the long imaging times and patient compliance.

Although promising preclinical data are available for several decades on CCK2R imaging in MTC [26], few clinical studies in human subjects were performed. A compound very similar to [^111^In]In-CP04, namely [^177^Lu]Lu-PP-F11N, was evaluated in a phase 0 study (LUMED) as a potential theranostic radiopharmaceutical in 6 patients with advanced MTC. Favourable pharmacokinetics and tumour targeting properties with low administered activity (1 GBq, 79 μg) were demonstrated for the compound, with average absorbed doses of 0.88, 0.42, 0.11, and 0.028 Gy/GBq to the tumour lesions, stomach wall, kidneys, and bone marrow, respectively [17]. Promising results led to an extension of LUMED into a phase I trial, where dose escalation and repeated administration in three patients was well tolerated, with comparable absorbed doses to the tumour lesions and moderate biochemical response (Ct, CEA) [27]. While the absorbed dose to the kidneys was comparable to the projected ^177^Lu absorbed dose for CP04 (0.12 Gy/GBq with concomitant Gelofusine infusion), our results showed significant effect of Gelofusine in reducing the radiation exposure of kidney parenchyma in contrast to the LUMED study. The reason for the discrepancy is unclear as the compounds are structurally, pharmacokinetically, and pharmacodynamically similar; the different half-lives of the radionuclides used and therefore different imaging time-points for dosimetry assessment, as well as small number of patients included in both studies, may have contributed to the observed differences and require further investigation.

Theranostic compounds labelled with positron-emitting radionuclides offer several advantages over single-photon labelled alternatives, including inherent tomographic imaging, improved sensitivity, higher spatial resolution, and shorter imaging time, as already demonstrated for somatostatin receptor targeting in neuroendocrine tumours [28–31]. Labeling of peptides with generator-produced ^68^Ga is one common pathway toward clinical application and was also attempted with CCK2R-targeting compounds. A comparison with conventional molecular imaging typically revealed increased lesion-to-background contrast and improved lesion detection [32, 33]. Improved sensitivity and whole-body assessment render imaging with CCK2-targeting PET probes ideal in the setting of MTC restaging after biochemical relapse as well as initial staging [34], where they may eventually replace alternative imaging methods and allow either targeted locoregional therapeutic approaches or initiation of systemic therapies. However, their most important advantage is undeniably the potential theranostic prospect. Currently, therapeutic options for advanced, metastatic MTC are limited. Kinase inhibitors are the mainstay of management with acceptable response rates, but are related to frequent adverse effects requiring reduction or cessation of treatment; the yield of combination chemotherapy is poor [5, 35]. PRRT with ^177^Lu/^90^Y radiolabelled somatostatin analogues is a viable therapeutic option in a minor proportion of patients due to the low density of somatostatin receptor expression [36]. Nevertheless, in appropriately selected patients, it was shown to be effective and well tolerated [37]. It is reasonable to assume that the typically high density of CCK2 receptor expression on MTC lesions will enable successful implementation of targeted radionuclide therapy, as recently demonstrated in a limited patient set [27]; further clinical studies with emerging CCK2 receptor ligands are warranted [33, 38].

## Conclusion

Administration of [^111^In]In-CP04 in patients with recurrent MTC is safe, even when high peptide mass is administered in advanced cases with extensive disease. Furthermore, its diagnostic performance surpassed that of conventional imaging in this study and may be superior to currently available molecular imaging approaches. Favourable biodistribution and projected target organ doses when labelled with the therapeutic radionuclide ^177^Lu provide an impetus for initiation of comparative clinical studies of the diagnostic performance of [^111^In]In-CP04 in patients with confirmed or suspected metastatic MTC as well as for the initiation of clinical trials with the aim of investigating the safety and tolerability profile as well as the efficacy of targeted radionuclide therapy with [^177^Lu]Lu-CP04.
